# Proteomic profiling of retinoblastoma by high resolution mass spectrometry

**DOI:** 10.1186/s12014-016-9128-7

**Published:** 2016-10-26

**Authors:** Ravikanth Danda, Kalaivani Ganapathy, Gajanan Sathe, Anil K. Madugundu, Sharavan Ramachandran, Uma Maheswari Krishnan, Vikas Khetan, Pukhraj Rishi, T. S. Keshava Prasad, Akhilesh Pandey, Subramanian Krishnakumar, Harsha Gowda, Sailaja V. Elchuri

**Affiliations:** 1 Department of Ocular Pathology, Vision Research Foundation, Sankara Nethralaya, Chennai, Tamilnadu 600006 India; 2 Department of Nano-Biotechnology, Vision Research Foundation, Sankara Nethralaya, Chennai, Tamilnadu 600006 India; 3 Shri Bhagwan Mahavir Vitreoretinal Services and Ocular Oncology Services, Medical Research Foundation, Sankara Nethralaya, Chennai, Tamilnadu 600006 India; 4 Institute of Bioinformatics, International Technology Park, Bangalore, Karnataka 560066 India; 5 Centre for Nanotechnology and Advanced Biomaterials, Shanmugha Arts, Science, Technology and Research Academy University, Tanjore, Tamilnadu India; 6 McKusick-Nathans Institute of Genetic Medicine, Johns Hopkins University School of Medicine, Baltimore, MD 21205 USA; 7 Department of Pathology, Johns Hopkins University School of Medicine, Baltimore, MD 21205 USA; 8 Department of Oncology, Johns Hopkins University School of Medicine, Baltimore, MD 21205 USA; 9 Department of Biological Chemistry, Johns Hopkins University School of Medicine, Baltimore, MD 21205 USA

## Abstract

**Background:**

Retinoblastoma is an ocular neoplastic cancer caused primarily due to the mutation/deletion of RB1 gene. Due to the rarity of the disease very limited information is available on molecular changes in primary retinoblastoma. High throughput analysis of retinoblastoma transcriptome is available however the proteomic landscape of retinoblastoma remains unexplored. In the present study we used high resolution mass spectrometry-based quantitative proteomics to identify proteins associated with pathogenesis of retinoblastoma.

**Methods:**

We used five pooled normal retina and five pooled retinoblastoma tissues to prepare tissue lysates. Equivalent amount of proteins from each group was trypsin digested and labeled with iTRAQ tags. The samples were analyzed on Orbitrap Velos mass spectrometer. We further validated few of the differentially expressed proteins by immunohistochemistry on primary tumors.

**Results:**

We identified and quantified a total of 3587 proteins in retinoblastoma when compared with normal adult retina. In total, we identified 899 proteins that were differentially expressed in retinoblastoma with a fold change of ≥2 of which 402 proteins were upregulated and 497 were down regulated. Insulin growth factor 2 mRNA binding protein 1 (IGF2BP1), chromogranin A, fetuin A (ASHG), Rac GTPase-activating protein 1 and midkine that were found to be overexpressed in retinoblastoma were further confirmed by immunohistochemistry by staining 15 independent retinoblastoma tissue sections. We further verified the effect of IGF2BP1 on cell proliferation and migration capability of a retinoblastoma cell line using knockdown studies.

**Conclusions:**

In the present study mass spectrometry-based quantitative proteomic approach was applied to identify proteins differentially expressed in retinoblastoma tumor. This study identified the mitochondrial dysfunction and lipid metabolism pathways as the major pathways to be deregulated in retinoblastoma. Further knockdown studies of IGF2BP1 in retinoblastoma cell lines revealed it as a prospective therapeutic target for retinoblastoma.

**Electronic supplementary material:**

The online version of this article (doi:10.1186/s12014-016-9128-7) contains supplementary material, which is available to authorized users.

## Background

Retinoblastoma (RB) is an intraocular cancer found in children and its incidence is approximately estimated to be 1 in 20,000 live births [1] and widely known to affect children under 5 years but also rarely reported in adults. It occurs due to the inactivation of both alleles of retinoblastoma (RB1) gene located at the 13q14 region of chromosome 13 [2, 3]. Abnormality/loss of RB1 gene initiates retinoma and genomic instability which primarily leads to RB [4]. These gene mutations of Rb1 when occur in germinal cells results in germinal RB (hereditary) and when the mutations are restricted to somatic cells lead to sporadic RB (non-hereditary). There are very few protein expression profiling studies on RB. In our previous study, we compared RB primary tumor with that of control retina using a two-dimensional (2DE) electrophoresis and mass spectrometry approach. We identified 27 differentially expressed proteins of which 16 were up-regulated and 11 were downregulated [5]. We could only probe a small number of relatively abundant proteins in our 2DE based proteomics approach. The gel free differential protein expression profiling using iTRAQ based quantitative proteomics strategy has emerged as a viable alternative to 2DE based proteomic approach [6]. This strategy coupled with highly sensitive mass spectrometers allows for proteomicprofiling of thousands of proteins in an experiment.

Isobaric tags for relative and absolute quantitation (iTRAQ) is a method which labels primary amines in peptides and offers multiplexing capability [7]. The approach relies on isobaric tags which upon fragmentation in the MS/MS gives rise to unique reporter ions. We used 4-plex iTRAQ reagents that yield reporter ions at *m*/*z* 114,115,116 and 117, [8]. Reporter ion intensities provide a measure of differential abundance of peptides that can be used to deduce differences in protein expression levels across multiplexed samples. In the present study, we report the first comprehensive proteomic signature using high resolution LC–MS/MS for comparative screening of RB.

## Methods

### Sample collection

The present study was conducted at Medical research and Vision research foundation, Sankara Nethralaya, India and was approved by the institutional ethics board. Control retina was collected from C.U. SHAH eye bank, Sankara Nethralaya in the age group of 18–28 years from the donated eye globes for corneal transplantation and were without known concomitant Ocular diseases. Tumor tissues were collected with informed consent. When the enucleated eye globe was sent for histopathological examination, a part of the tumor was collected for proteomic study and the other part was used for histopathological studies (Additional file 1: Table S1). The collected tumors were snap frozen in liquid nitrogen and transferred to −80 °C until used for proteomic analyses.

The tissues were thawed on ice, resuspended in lysis buffer (0.5 % SDS buffer) and sonicated on ice for 2–3 cycles. The samples were centrifuged at 12,000 rpm for 10 min at 4 °C. The supernatants were collected and stored at −80 °C until further use. For proteomic analysis, equal amount of protein from normal retinas (n = 5) was pooled and compared with a pooled RB samples (n = 5).

### iTRAQ labeling

Isobaric tags for relative and absolute quantitation (iTRAQ) labeling was performed as per the manufacturer’s protocol. Briefly, 100 µg of pooled retina and tumor proteins were incubated with reducing agent [tris (2-carboxyethyl) phosphine] at 60 °C for 60 min. Methylsulfenylation of cysteine residues was carried out using cysteine blocking reagent (methyl methanethiosulfonate) for 10 min at room temperature as per manufacturer’s protocol. Tryptic digestion was performed overnight at 37 °C in sequencing grade trypsin (Promega, San Luis Obispo, CA, USA). In order to maintain a technical replicate, tryptic digests of normal retina was split in equal halves and labeled with iTRAQ reagents yielding reporter ions at 114 and 115 *m*/*z*. Similarly, tryptic digests from RB sample were split in equal halves and labeled with iTRAQ reagents yielding reporter ions at 116 and 117 *m*/*z*. All the samples were pooled, vacuum dried and stored at −20 °C until further use.

### SCX fractionation

The labeled peptides were reconstituted in 1 mL of solvent A [5 mM KH_2_PO_4_, 25 % acetonitrile (ACN), pH 2.7] and separated using Agilent 1200 series offline HPLC. Peptide fractionation was carried out using strong cation exchange chromatography. Peptides were eluted using a linear gradient of solvent B (350 mM of potassium chloride in solvent A) at a flow rate of 250 µL/min for 50 min. Consecutive fractions with relatively less peptides (based on UV absorbance at 280 nm) were pooled and a total of 18 fractions were prepared for LC–MS/MS analysis. The peptides were vacuum dried and reconstituted in 0.1 % trifluoroacetic acid (TFA). The samples were desalted using C18 zip tips before LC–MS/MS analysis.

### LC–MS/MS analysis

LC–MS/MS analysis was carried out on LTQ-Orbitrap Velos mass spectrometer (Thermo Scientific, Bremen, Germany) interfaced with proxeon Easy nanoLC system. The peptide samples were enriched on a trap column (75 µm × 2 cm) at a flow rate of 3 µL/min and resolved on an analytical column (75 µm × 10 cm) at a flow rate of 350 nL/min. These were eluted by using a linear gradient of 7–30 % of solvent B (90 % ACN and 0.1 % formic acid) for 100 min. The MS analysis was performed on Orbitrap mass analyzer in a data dependent manner with full scans acquired at a mass resolution of 60,000 at 400 *m*/*z*. 20 most intense precursor ions were chosen for fragmentation in each cycle. MS/MS fragmentation was carried out using high energy collision dissociation with 41 % normalized collision energy at a mass resolution of 15,000 at 400 *m*/*z*. The isolation window was set to 2 *m*/*z*. The precursor ions that were fragmented were dynamically excluded for 45 s. Full scans were acquired with AGC target value of 100,000 and for FT MS/MS it was set at 50,000 and maximum accumulation time was 300 and 200 ms, respectively. The lock mass was enabled for accurate mass measurement.

### Protein identification and quantitation

**Fig. 1:**
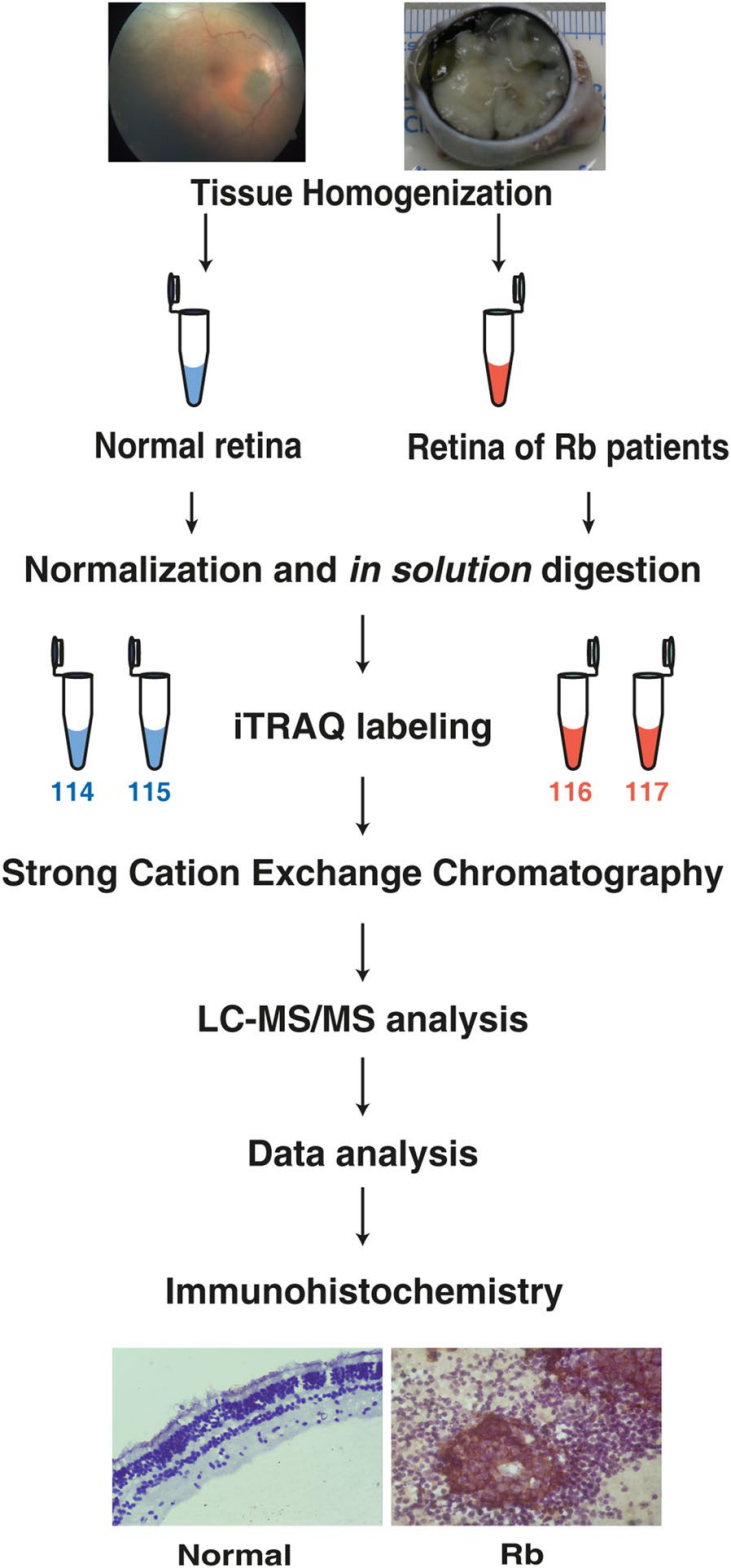
Schematic representation of work flow for the sample preparation and data analysis of total proteome

The obtained MS data was analyzed using Proteome Discoverer (version 1.3). The peptide search work flow includes spectrum selector followed by reporter ion quantitation (Fig. 1). The spectra were searched using MASCOT and SEQUEST search algorithm against NCBI RefSeq database version 65 containing 34,454 proteins. The search parameters included trypsin as the enzyme with one missed cleavage allowed, methylsulfenylation of cysteine, iTRAQ modifications at N-terminus of peptide and lysine as static modifications. Oxidation of methionine was set as dynamic modification. Precursor and fragment mass tolerance was set at 20 ppm and 0.1 Da, respectively. The protein and peptide data were extracted using high peptide confidence and top one peptide rank filters. The false discovery rate (FDR) was calculated by searching the peptide sequences against a decoy database and cut off of <1 % was used for identified peptides.

### Bioinformatic analysis

Bioinformatic analysis and annotations of the obtained protein list was carried out based on their localization, biological process and molecular function as per human protein reference database (HPRD) [9], which is in compliance with the gene ontology standards. Pathway analysis was performed by ingenuity pathway analysis (Ingenuity Systems, Redwood City, CA, USA).

### Immunohistochemistry

A select set of differentially expressed proteins in RB were validated in individual tumor tissues (n = 15). Immunohistochemical labeling (IHC) of CHGA, AHSG, IGF2BP1 RACGAP1 and MDK was performed on paraffin wax embedded tumor sections. Deparaffinization and antigen retrieval was followed as described previously [5]. The antibodies and their dilutions are as follows: anti-CHGA polyclonal antibody at 1:25, polyclonal antibody at 1:10, anti-AHSG polyclonal antibody at 1:25, anti-RACGAP1 polyclonal antibody at 1:25 anti-IGF2BP1 polyclonal antibody at 1:25, anti-MDK polyclonal antibody at 1:150 for IHC applications. All the antibodies were purchased from Pierce antibodies and raised in rabbit against human proteins. Staining for all the five proteins were observed with anti-rabbit IgG-Poly-HRP using the NovoLink Max Polymer (Leica biosystems, Germany) detection system according to the manufacturer’s protocol. The protein expression was calculated and the tumors were grouped into Group I (1–33 %), Group II (34–67 %), Group III (68–100 %) based on their staining intensities. The intensity was given ‘–’ as negative, rank ‘±’ for dull and rank ‘+’ for intense staining. Overall distribution and staining pattern of the tissues was ranked from 0 to 6 that were obtained by multiplying the group and the staining intensity. The staining intensities were ranked one for negative, ranked two for dull and ranked three for intense staining (Additional file 1: Table S2).

### Cell culture

RB cell line Y79 was obtained from Riken Bio Resource center (Japan). Y79 cells were cultured in ATCC modified RPMI 1640 (Invitrogen, USA) media containing sodium pyruvate supplemented with 10 % fetal bovine serum (FBS) (Invitrogen, USA) at 37 °C in 5 % CO_2_ humidified incubator.

### mRNA knockdown studies

For evaluating the role of IGF2BP1 over expressed protein in tumor progression, siRNA based knock down study of IGF2BP1 protein was performed in Y79 RB cell line. The siRNA was delivered into the cells using Lipofectamine 2000 (Invitrogen, USA) according to the manufacturer’s protocol. The IGF2BP1 siRNA (5′ CCGGGAGCAGACCAGGCAA3′) was obtained from Dharmacon, Thermo Scientific, Pittsburg, PA, USA. Transfections were performed in 50 pm/µL of IGF2BP1 siRNA and scrambled siRNA. The cells were seeded 24 h prior to the experiment and were incubated for 48 h post transfection. The cells were collected at 3000 rpm for 10 min at 4 °C. The collected cells were used for the downstream processes involving western blot and real-time PCR.

### Real-time PCR

Total RNA was isolated using Trizol reagent (Invitrogen, USA). cDNA synthesis was carried out using high capacity reverse transcriptase kit (Applied Biosystems, USA) as per the manufacturer’s protocol. Ct values for the target genes were calculated and normalized against GAPDH housekeeping gene. The primers used for the real-time PCR were IGF2BP1 FP 5′ TAGTACCAAGAGACCAGACCC 3′ RP 5′ GATTTCTGCCCGTTGTTGTC 3′ GAPDH FP 5′ GCCAAGGTCATCCATGACAAC 3′ RP 5′ GTCCACCACCCTGTTGCTGTA 3′. The fold changes of the genes were expressed in the log2 relative units. The PCR products were detected using ABI PRISM 7500 detection system and analysis was done on ABI PRISM 7500 SDS software (Applied Biosystems, USA).

### Western blot analysis

For western blot analysis, the IGF2BP1siRNA was transfected into Y79 as previously described. The cells were collected and washed twice with PBS. The washed cells were re-suspended in RIPA buffer (R 0278, Sigma, USA). The lysed cells were spun at 12,000 rpm for 5 min at 4 °C. Protein estimation was carried out using BCA reagent (Thermo Scientific, USA) as per the manufacturer’s protocol. Equal amount of protein (50 µg) was resolved using polyacrylamide gel electrophoresis and transferred on to a nitrocellulose membrane. The membrane was incubated with IGF2BP1 primary antibody at 4 °C overnight. The membrane was washed and probed with mouse anti-rabbit (Sigma Aldrich, USA) secondary antibody for 2 h at room temperature. After the incubation time, the membrane was developed with TMB H_2_O_2_ (Bangalore Genei, India), scanned and documented. The membrane was stripped with stripping solution and re-probed with β-actin primary antibody (Sigma Aldrich, USA). The membrane was washed twice with PBS and probed with rabbit anti-mouse (Sigma Aldrich, USA) secondary antibody for 2 h. The membrane was developed with TMB H_2_O_2_, scanned and documented.

### Proliferation assay

3-(4,5-Dimethylthiazol-2-yl)-2, 5-diphenyltetrazolium bromide (MTT) assay was performed to evaluate the percentage of viable cells in the siRNA treated cells. Briefly, the cells were transfected with the siRNA and incubated for 48 h. Post incubation, the cells were replaced with 10 % of MTT in serum containing media and incubated for 4 h at 37 °C. The formazan crystals were confirmed, dissolved in DMSO and absorbance was read at 570 nm using Spectramax M5 spectrophotometer (Molecular devices, USA). Data was expressed as mean ± SD of 3 independent experiments and each experiment was done in triplicates.

### Wound healing assay

To evaluate the migration of Y79 cells, wound healing assay was performed. The cells were seeded 1 day prior to the treatment with siRNA in a poly-l-lysine coated plate. The cells were transfected with siRNA and a wound was created with a tip. The cells were documented at 0 h and incubated for 24 h and again documented to assess the migration of cells using Axio Vision phase contrast microscope.

### Data availability

The mass spectrometry proteomics data have been deposited to the ProteomeXchange Consortium [10] via the PRIDE partner repository with the dataset identifier PXD002774.

## Results and discussion

**Fig. 2:**
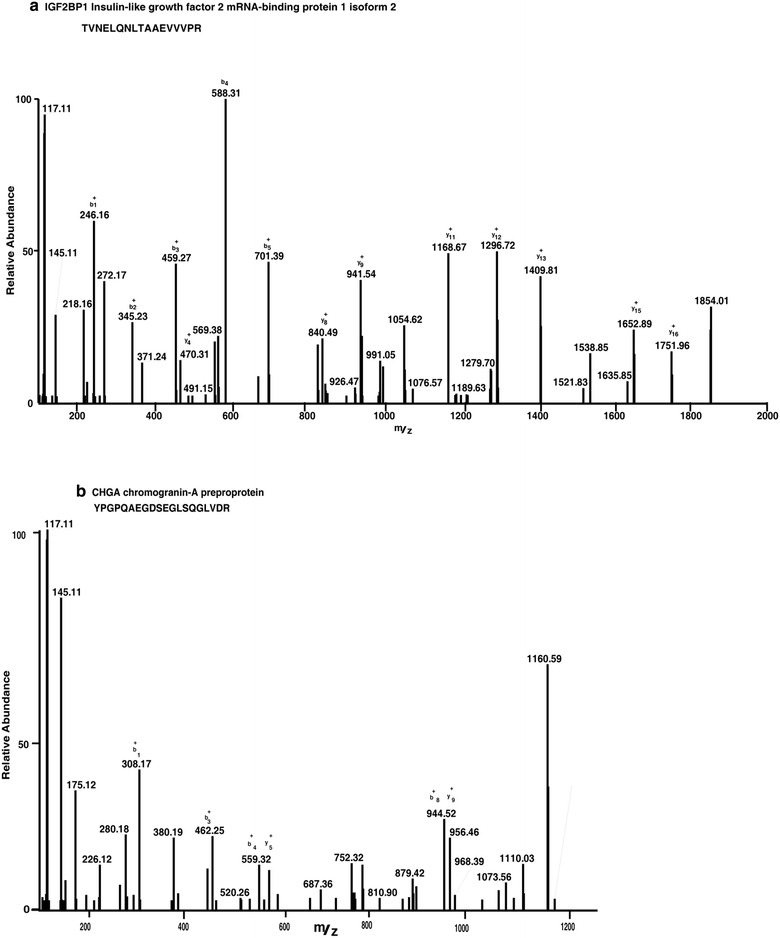
MS/MS spectra of the peptides with their reporter ions for the over expressed proteins in retinoblastoma. Relative intensities of reporter ions for **a** IGF2BP1 and **b** CHGA

**Fig. 3:**
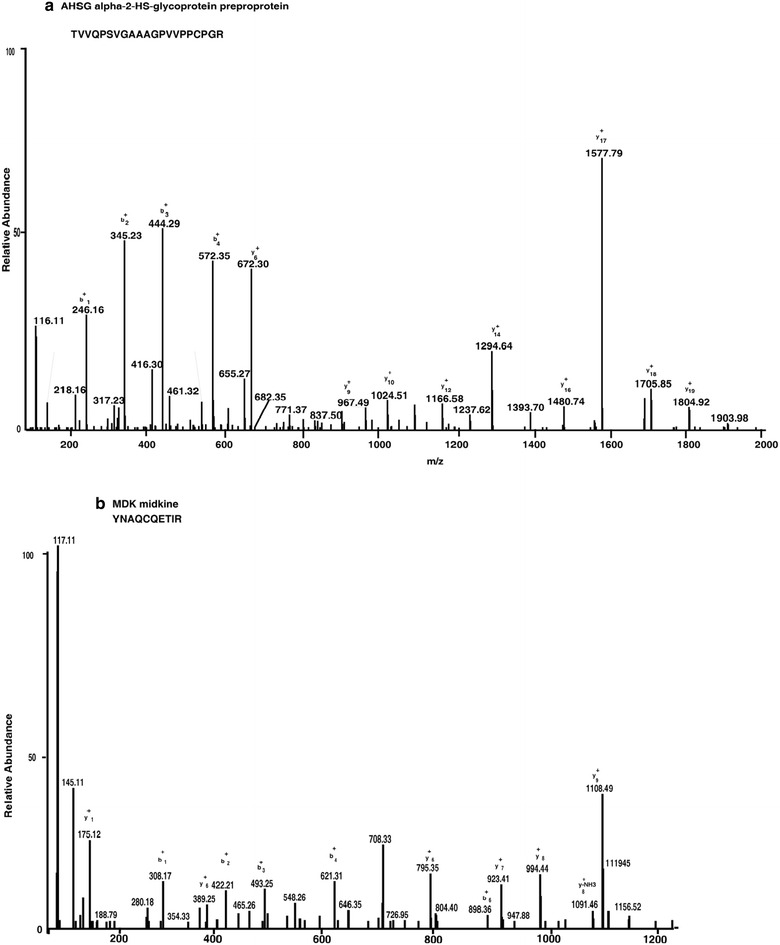
MS/MS spectra of the peptides with their reporter ions for the over expressed proteins in retinoblastoma. Relative intensities of reporter ions for **a** AHSG and **b** MDK

We have analyzed the total proteome of RB isolated from the pooled clinical samples which reduces the individual tumor variation and leads to increase in confidence of target molecule identification. The clinicopathological features of the tumours used in the proteomic study include samples below 4 years (Additional file 1: Table S1). We performed iTRAQ labeling and LC–MS/MS to generate the differentially expressed proteins. We identified 3671 and quantified 3587 proteins, out of which, 899 proteins were differentially expressed with a fold change of ≥2 (Additional file 2). These 899 (402 up-regulated and 497 down-regulated) differentially expressed proteins were taken for further analysis. Differentially expressed proteins include spleen tyrosine kinase (SYK), stathmin1 (STMN1) and vimentin (VIM) that have been previously reported to be overexpressed in RB [5, 11]. In addition, we identified several novel proteins and validated CHGA, AHSG IGF2BP1 and RACGAP1 that were previously unreported in RB by IHC. Representative MS/MS spectra of differentially expressed proteins are shown in Figs. 2, 3.

### Overlap of proteomic data with previously published datasets

**Fig. 4:**
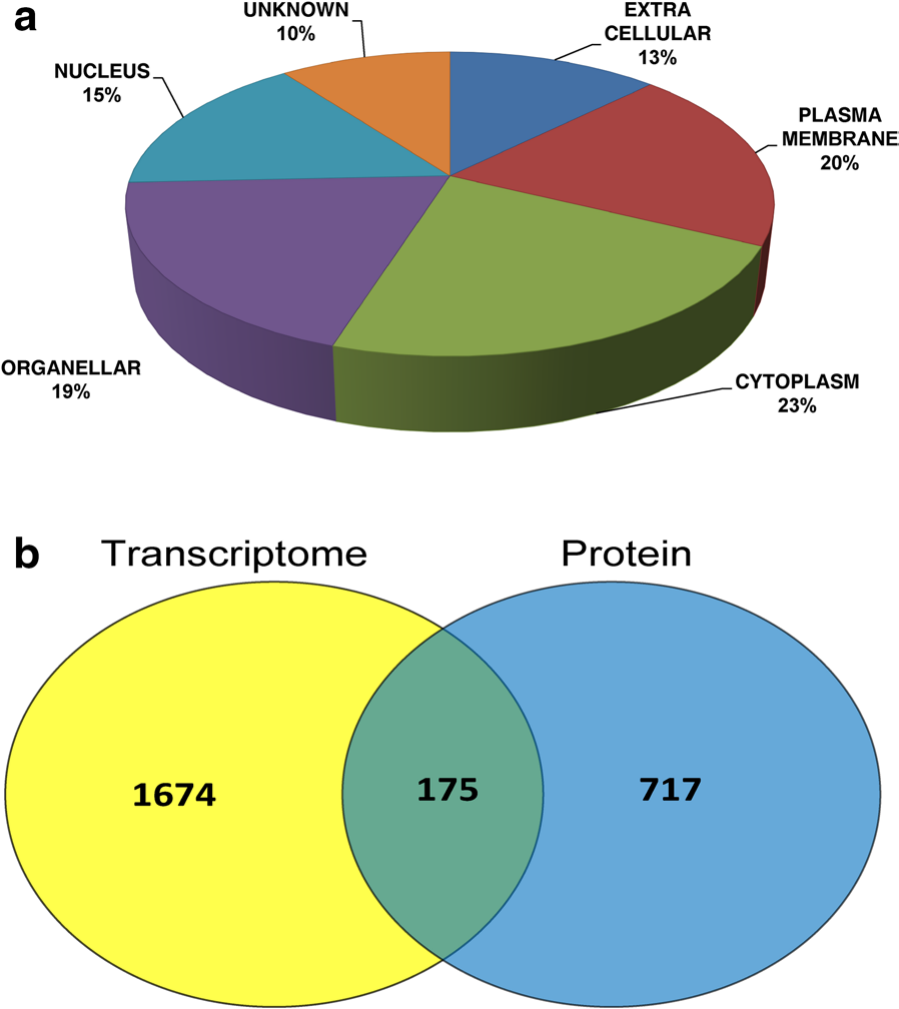
**a** Sub-cellular classification of differentially regulated proteins in Retinoblastoma based on annotations from human proteome reference database. **b** Comparison of deregulated proteins involved in present study with previously reported transcriptome data of retinoblastoma. A total of 175 proteins showed positive correlation with the transcriptome

The observed 899 differential proteins were compared with published transcriptome dataset [12]. We observed that 175 proteins were overlapped with the published transcriptome profile. The overlapped dataset (Fig. 4) showed 100 proteins were up-regulated and 73 proteins were down-regulated which indicates that these proteins are transcriptionally regulated.

### Previously reported up-regulated proteins

We then examined the presence of overexpressed proteins reported previously in RB. Cellular retinoic acid binding protein 2 (CRABP2) is a cytoplasmic protein, which translocates to nucleus upon ligand binding. CRABP2 is involved in cell proliferative activity by activating PPARβ/δ and FABP5 proteins [13]. In our data, we observed that FABP5 was not up-regulated suggesting an alternative mechanism for the activation and overexpression of CRABP2 in RB tumors. CRABP2 is known to be overexpressed in variety of cancers such as breast and ovarian cancers [14, 15]. The siRNA based knockdown study of CRABP2 in head and neck squamous cell carcinoma showed decrease in tumour cell invasion [16]. Kinesin like protein 11 (KIF11) belongs to family of protein Kinesins and are classified into 14 subfamilies (1–14) based on their phylogenetic analysis of motor domain. It is involved in cell division and intracellular vesicle and organelle transport. In one of the earlier studies on mRNA profiling of RB it was found to be 15-fold upregulated [12]. In our present quantitative proteomic profiling, KIF11 was threefold upregulated (Additional file 2). Knockdown studies of KIF11 by RNAi and antibodies showed activation of mitotic arrest, inhibition of spindle formation and apoptosis in the tumor cells [17]. SYK, is a 72 kDa protein localized to cytoplasm. It is a non-receptor kinase involved in signal transduction and plays an important role in regulation of immunomodulatory signaling and involved in several hematopoietic malignancies. In an earlier study by Zhang et al. [11], overexpression of SYK in RB was involved in cell survival. Knockdown studies by shRNA and small molecule inhibitor revealed that SYK inhibition leads to increased apoptosis in RB cell lines and orthotopic xenografts models respectively. In concordance with the earlier study a 12-fold upregulation of SYK was observed in our present dataset.

### Bioinformatics analysis

Sub-cellular classification of the differentially expressed proteins was done using HPRD [9] (Fig. 4). In our present study, of the 899 differentially expressed proteins, 187 are localized in cytoplasm, 162 in plasma membrane, 128 in the nucleus, 104 proteins are putative extracellular proteins and 160 are localized to other organelles. A few differential proteins do not have the localization information in the literature.

### Pathway analysis

In order to understand the biological significance of these molecules, we analyzed these proteins using Ingenuity pathway knowledge base and identified the Canonical pathways, networks, molecular and cellular processes, diseases and disorders that are relevant to the dataset. The top five canonical pathways identified include mitochondrial dysfunction, LXR/RXR activation, acute phase response signaling, complement system and photo transduction pathway. Neurological diseases, skeletal and muscular disorders, cancer, hereditary disorders and physiological disorders were major diseases and disorders identified. The top molecular and cellular processes identified include lipid metabolism, molecular transport, small molecule biochemistry, nucleic acid metabolism, DNA replication, recombination and repair. Lipid metabolism and mitochondrial dysfunction are the top most deregulated pathways. In many cancers, lipid synthesis is key metabolic process upregulated for cancer progression and metastasis. Increase in the concentration of lipids leads to the formation of lipid droplets which are required for the membranes synthesis, cellular metabolism and energy production [18]. Mitochondrial dysfunction in the present study included down regulation of proteins from Complex I, Complex III, Complex IV and Complex V which are responsible for the generation of energy in the cell. Complex I (CI) is the first component that regulates the production of reactive oxygen species (ROS) and ATP generation [19]. In the absence/defective CI there is a reduction in the ATP generation which effects the cell survival, which could be compensated by increase in the lipid synthesis and metabolism as energy source in RB.

### Validation of novel proteins identified in retinoblastoma by immunohistochemistry

**Fig. 5:**
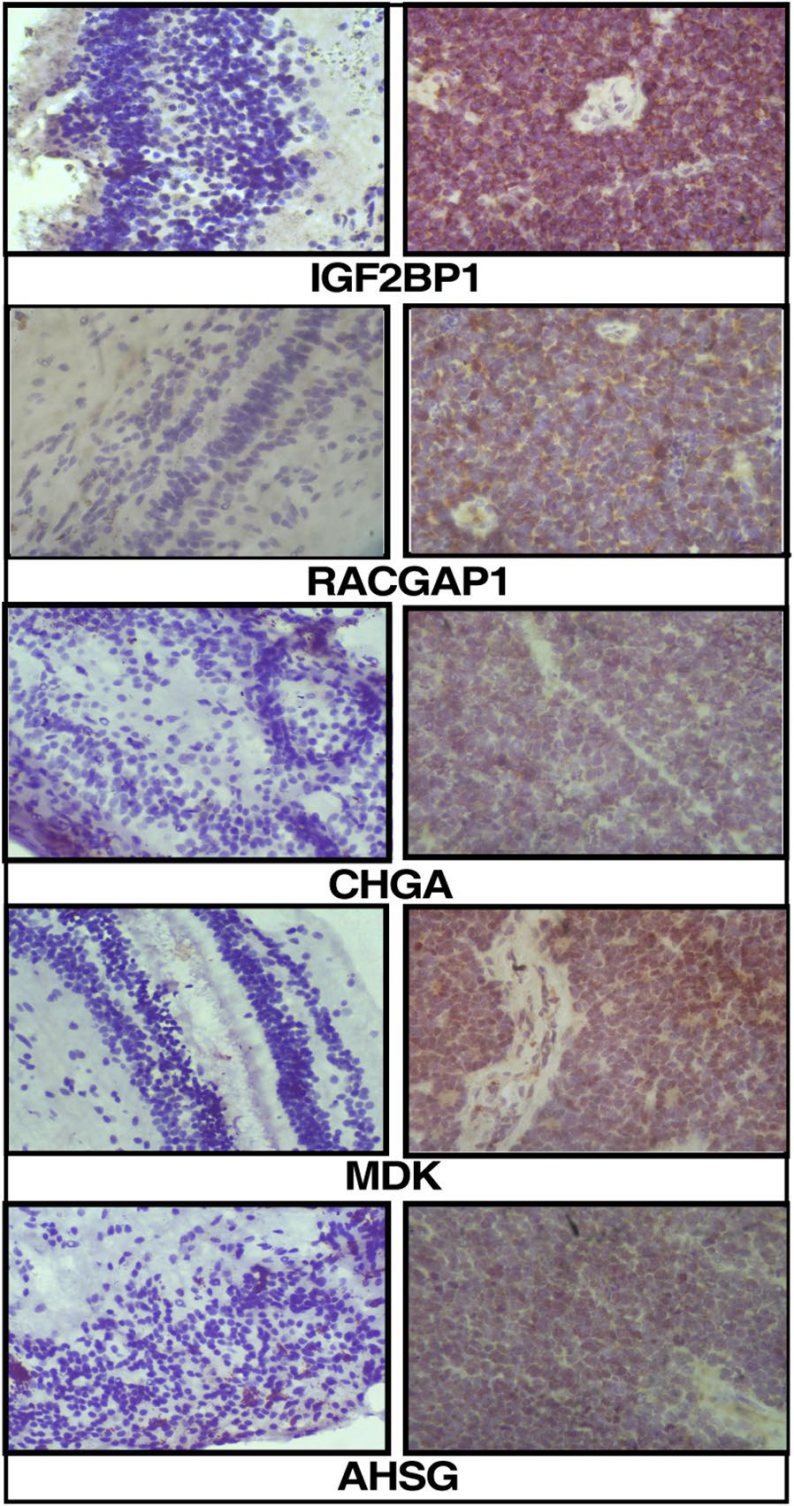
Immunohistochemistry of selected proteins IGF2BP1, RACGAP1, CHGA, MDK and ASHG in retinoblastoma primary tumors and non-neoplastic control retina

In the present study, we identified 559 novel proteins with no previous report of differential expression in RB compared to retina. A partial list of novel proteins identified and their corresponding fold change are given in Table 1. Some of the proteins identified in our dataset were further evaluated by IHC (Fig. 5; Table 1). The proteins selected for IHC validation were based on magnitude of overexpression and novelty in the context of RB, involvement of proteins in tumor progression. CHGA, AHSG, RACGAP1, IGF2BP1 and MDK were selected for validation studies on formalin fixed paraffin embedded tissue sections. These proteins were overexpressed more than twofold compared to non-neoplastic retina. The staining intensity and distribution is summarized in Table 2. Fifteen tumors and three normal retinas were taken for IHC analysis. All 15 tumors showed positivity for all the antibodies. Nearly 50 % of tumor sections showed strong staining for CHGA and the remaining tumors showed moderate staining. 90 % of the tumor sections showed strong staining for AHSG, RACGAP1, IGF2BP1 and MDK the remaining tumors showed moderate staining with the antibodies. All the non-neoplastic retinas showed no staining for these proteins using the same antibodies (Additional file 1: Table S2).

**Table 1:** A partial list of novel proteins identified in retinoblastoma

Gene symbol	Protein	Peptides	Fold change Tumor/normal
HIST1H1B	Histone H1.5	6	16.9
CHGA	Chromogranin-A	4	15.6
IGF2BP1	Insulin-like growth factor 2 mRNA-binding protein 1	2	13.1
KRT17	Keratin, type I cytoskeletal 17	9	12.8
KRT16	Keratin, type I cytoskeletal 16	17	12.8
LOC102723407	Putative V-set and immunoglobulin domain-containing-like protein IGHV4OR15-8-like	2	12.7
RACGAP1	Rac GTPase-activating protein 1	1	12.3
SYK	Tyrosine-protein kinase SYK isoform Syk(S)	2	12.1
KRT6C	Keratin, type II cytoskeletal 6C	18	11.6
AHSG	Alpha-2-HS-glycoprotein	9	11.3
CHAMP1	Chromosome alignment-maintaining phosphoprotein 1	3	11.2
APOA2	Apolipoprotein A-II	3	10.9
DDX49	Probable ATP-dependent RNA helicase DDX49	1	10.6
LAMA3	Laminin subunit alpha-3	1	10.4
IGFALS	Insulin-like growth factor-binding protein complex acid labile subunit	1	10.2
HMGA2	High mobility group protein HMGI-C isoform a	2	10.1
MCM4	DNA replication licensing factor MCM4	16	10.0
KPNA2	Importin subunit alpha-1	2	9.4
DDB2	DNA damage-binding protein 2	1	9.4
IGF2BP3	Insulin-like growth factor 2 mRNA-binding protein 3	7	9.3
MDK	Midkine isoform b	1	9.0
APOA4	Apolipoprotein A-IV	14	8.5
IGLL5	Immunoglobulin lambda-like polypeptide 5	5	8.4
MCM6	DNA replication licensing factor MCM6	15	8.3

**Table 2:** Immunohistochemistry scoring of 15 tumors and three control retina the details of scoring is described in “Methods” section

Protein	Proteomic data	Sample	No of cases showing expression	Staining intensity		
				0 (−)	1 (±)	2 (+)
CHGA	Up-regulated	Case	15/15		9	6
		Control	3	0		
AHSG	Up-regulated	Case	15/15		3	12
		Control	3	0		
RACGAP1	Up-regulated	Case	15/15		2	13
		Control	3	0		
MDK	Up-regulated	Case	15/15		3	12
		Control	3	0		
IGF2BP1	Up-regulated	Case	15/15		3	12
		Control	3	0		

#### CHGA

Chromogranin A is a glycoprotein of 439 amino acids and belongs to the family of secretogranin [20]. It is an extracellular matrix protein released with co-hormones from the neuroendocrine cells into the extracellular environment and then into circulation [21]. CHGA is the precursor molecule for many of the bioactive peptides like vasostatin I and II, chromacin, pancreastatin, parastatin, catestatin and WE-14 [22]. It inhibits the VEGF and thrombin induced endothelial cell permeability [23] and helps in controlling the angiogenesis and vascular leakage by inhibiting the TNFα elicited effect on endothelial cells. High expression of CHGA in RB might be involved in causing increased cell proliferation, migration and motility leading to tumor formation and increased angiogenesis for tumor growth. High expression of CHGA was observed in small cell lung cancer, neuroendocrine tumors, medullary carcinoma of thyroid and in neuroblastoma [24].

#### AHSG

Alpha-HS-glycoprotein also called as fetuin-A, is a serum glycoprotein synthesized and secreted by brain, kidneys and liver into serum [25]. However presence of the protein is identified in pancreatic ductal adenocarcinoma and hepatocellular carcinoma tissues [26, 27]. AHSG is a 51–67 kDa and belongs to cystatin family of proteins lacking protease-inhibitory capacity [28, 29]. It regulates and activates the PI3- kinase/AKT pathway, which is a downstream signaling molecule of TGFβ resulting in impaired tumor growth [30]. In one study on breast cancer, AHSG enhanced the formation of exosome, which helped in the cell adhesion in the metastatic cancer cells [31]. The cell adhesion in the presence of AHSG is mediated by annexins [32].

#### MDK

Midkine is a heparin binding growth factor involved in cell migration and proliferation. It is a 143 amino acid protein which includes secretory signal peptides yielding 13 kDa mature peptides [33]. The C-terminal domain of MDK consists of two clusters composed of lysine and arginine residues that are involved in the heparin binding activity [34]. The cell surface receptors for MDK includes ALK, LRP1, integrin, PTPζ, notch2, glypican2, neuroglycan of which glypican2 was found to be twofold upregulated in our study. MDK mediates cell proliferation by activating MEK1/2 and PI3K signaling pathways. In tumor cell lines, inhibition of apoptosis and mitogenic function mediated by MDK induced phosphorylation of MAP kinases, extracellular signal regulated kinases and PI3 kinases. In our present study, MDK showed a 9.5-fold change. This was further confirmed by IHC where we observed high staining intensity for MDK (Table 2). This protein is found to be overexpressed in many cancers such as oral, brain and cervical [35, 36].

#### IGF2BP1

The insulin-like growth factor-2 mRNA-binding protein 1 is a 58–66 kDa protein that belongs to highly conserved RNA binding protein family (RBP family). The RBP family consists of IGF2BP1, IGF2BP2 IGF2BP3 which shows 56 % amino acid identity and high similarity within the protein domains. IGF2BP1 and IGF2BP3 show a higher identity of 73 % with each other. These RNA binding proteins (RBPs) consists of two RNA-recognition motifs (RRMs) in the N-terminal region and four hnRNP-K homology (KH) domains in the C-terminal region [37]. These are localized to cytoplasm with granular in appearance and alternatively these are also localized to nucleus. IGF2BP1 showed higher expression in embryo and lower expression levels in adult organs with exceptions in reproductive organs [37]. IGF2BP1 transcriptional expression was negatively regulated by CTNNB1 and positively regulated by MYC [38]. In our study, negative feedback could be active as CTNNB1 is found to be twofold down regulated and IGF2BP1 was 14-fold up regulated whereas c-MYC remained unaltered. In one study it was shown that IGF2BP1 binds to MYC mRNA and inhibits its degradation in vitro but it was not observed in vivo [39]. Immunohistochemical staining pattern showed high expression of IGF2BP1 in cytoplasm in all RB tumors.

#### RACGAP1

Rac gap activating protein (RACGAP1) is a 63 kDa protein involved in the spindle fibre complex by interacting with KIF23 which are essential for cytokinesis [40] and also in the invasion/migration of cancer cells [41]. It is involved in the invasion/migration by activating PKB/AKT pathway. In an earlier study it was found that RACGAP1 knockdown resulted in reduction of cancer cell migration [42]. Ras GTPase activating like protein 1 (IQGAP 1) is a scaffolding protein with multiple protein interacting domains. It is involved in many functions such as cell–cell adhesion, cell polarization and directional migration. Recent studies showed that it interacts with phosphorylated RACGAP1 to form a complex and suppresses Rac and activates Rho A, stimulating cell invasion/migration. Knockdown studies of IQGAP1 showed reduction in invasion/migration in ovarian cancer cell lines [42]. We observed both the proteins were upregulated in our study, which could be the reason for the invasiveness of the RB tumor.

### Functional validation of overexpressed proteins

**Fig. 6:**
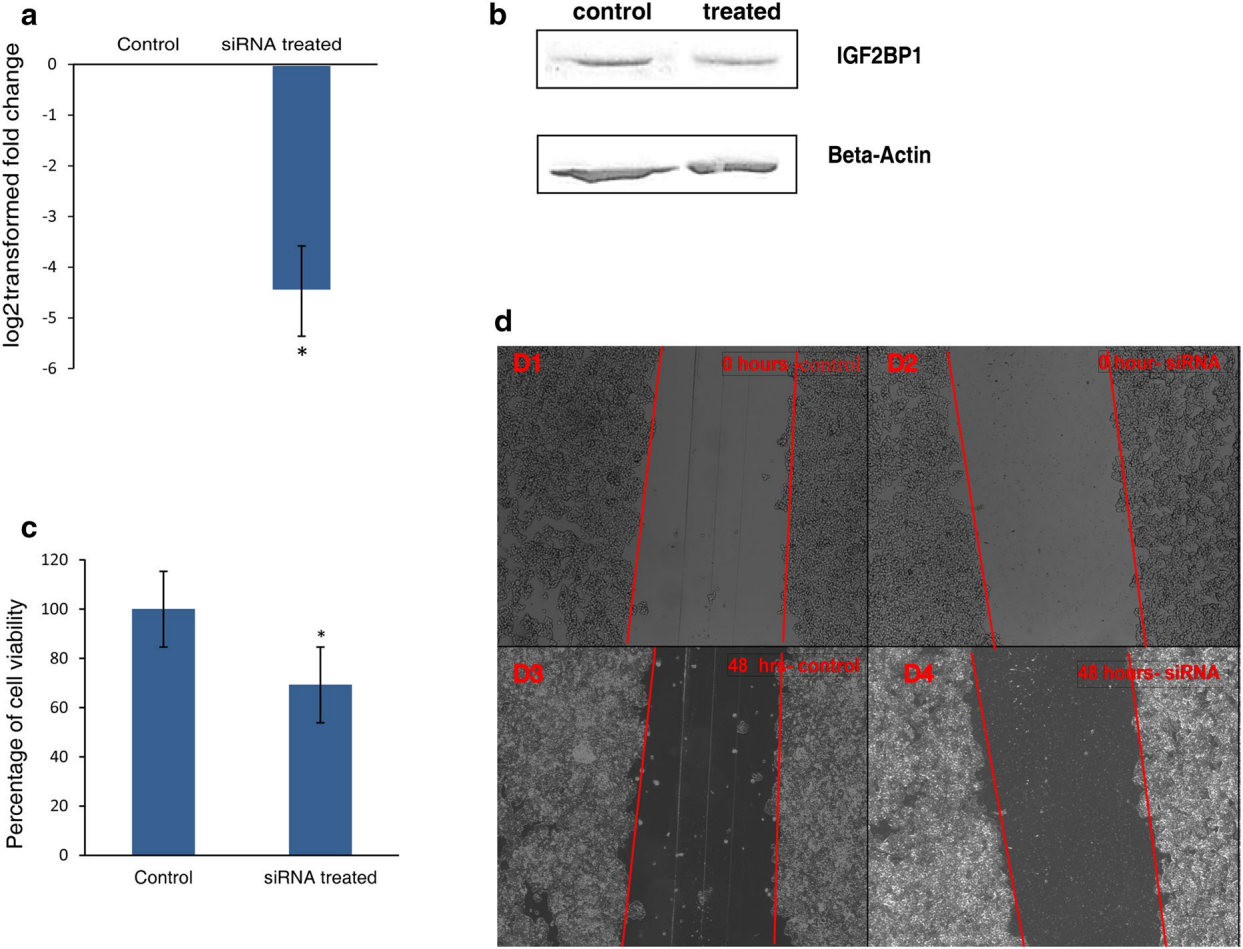
IGF2BP1 knockdown decreases Y79 RB cell proliferation and migration. **a** Comparison of mRNA expression of IGF2BP1 gene in siRNA untreated cells to treated cells. **b** Comparison of protein expression of IGF2BP1 in siRNA untreated to knockdown cells by western blot analysis. The *top band* shows IGF2BP1 expression and *bottom band* shows loading control, actin protein expression. **c** Comparison of percentage of cell viability in control and siRNA treated cells by MTT assay. **d** Comparison of migration of cells by wound healing assay in control (**d1**, **d3**) and siRNA treated cells (**d2**, **d4**). The *top figure* shows cell migration at 0 h and *bottom* shows figure cell migration at 48 h after the creation of the wound. *P < 0.05

To understand the involvement of the over-expressed proteins in cell proliferation, we performed functional knockdown of IGF2BP1 protein which is over-expressed in our study. Knockdown studies of the IGF2BP1 were performed by siRNA based RNA interference studies in Y79 RB cell lines. As we did not observe a significant difference of IGF2BP1 mRNA and protein levels between untreated and scrambled siRNA treated cells (data not shown), knockdown of IGF2BP1 studies were carried out with untreated Y79 cells as control. A 4.5-fold reduction in mRNA was observed in siRNA treated cells (Fig. 6a). Western blot analysis revealed reduced protein expression in treated when compared to control cells (Fig. 6b). We evaluated the role of this protein in cell proliferation/migration. Cell viability by MTT assay showed reduction in cell proliferation by 30–40 % in siRNA treated Y79 cells compared to untreated Y79 cells (Fig. 6c). Wound healing assay showed decreased cell migration in the cells treated with siRNA when compared to untreated Y79 cells at 48 h (Fig. 6d). These results indicate that reduction in the expression of IGF2BP1 leads to decreased proliferation and cell migration of RB cells indicating a novel potential therapeutic target. In hepatocellular carcinoma, stable knockdown of IGF2BP1 showed reduction in tumor migration and induction of apoptosis [43]. Overexpression of IGF2BP1 in rhabdomyosarcoma induced drug resistance [44]. The mechanism of cell proliferation and anti-apoptotic effect of IGF2BP1in RB needs further elucidation.

## Conclusions

This is the first comprehensive report of proteomic profile of RB tumor. The proteomic profile offered insights into proteins involved in tumor proliferation which were not reported previously. Functional validation of IGF2BP1 demonstrated its role in cancer cell proliferation/migration and offers a putative novel therapeutic target molecule for RB.

## Additional files

**Additional file 1: Table S1.** The clinicopathological features of the RB tumors from patients used in the study for the protein extraction and proteoomic analysis described in detail in the methods section.** Table S2.** The clinicopathological features of the RB tumors from patients used for the validation of the identified proteins using Immunohistochemistry along with the scores, described in detail in the methods section.

**Additional file 2.** List of proteins and corresponding peptides identified in the present study.

## Abbreviations

RB: retinoblastoma
IGF2BP1: insulin growth factor 2 mRNA binding protein 1
CHGA: chromogranin A
AHSG: fetuin A
RACGAP1: Rac GTPase-activating protein 1
MDK: midkine
IHC: immunohistochemistry
