# Supplementary material for: Proteomic profiling of retinoblastoma by high resolution mass spectrometry
Source: Clin Proteomics. 2016 Oct 26;13:29. doi: 10.1186/s12014-016-9128-7 (PMC5080735; doi:10.1186/s12014-016-9128-7)
Supplement: Supplementary file 1 — Additional file 1: Table S1. The clinicopathological features of the RB tumors from patients used in the study for the protein extraction and proteoomic analysis described in detail in the methods section. Table S2. The clinicopathological features of the RB tumors from patients used for the validation of the identified proteins using Immunohistochemistry along with the scores, described in detail in the methods section. [file 12014_2016_9128_MOESM1_ESM.docx]

Table S1 Clinicopathological features of RB tumors used for Immunohistochemistry

| **S.NO** | **AGE**  **/SEX** | **CLINICOPATHOLOGICAL FEATURES** | | **IGF2BP1** | | | |
| --- | --- | --- | --- | --- | --- | --- | --- |
|  |  | DIFFERENTIATION | INVASION  STATUS | %POSITIVE CELLS | STAINING  INTENSITY | SCORE | GROUP |
| 1 | 7 Mon/F | MD | CI <3mm .ON Inv –ve. | 60 | + | 4 | E |
| 2 | 1Y/M | PD | CI < 3mm. ON Inv –ve. | 60 | + | 4 | D |
| 3 | 6 Y/M | PD | CI >3mm. ON Inv –ve | 30 | ± | 1 | E |
| 4 | 1 Y/M | PD | CI <3mm. ON Inv –ve | 80 | + | 6 | D |
| 5 | 1 Y/F | WD | CI <3mm. ON Inv –ve | 80 | + | 6 | E |
| 6 | 1 Y/F | WD | CI <3mm, ON -ve | 50 | + | 4 | E |
| 7 | 10 Mon/F | WD | CI >3mm.ON Inv –ve. | 50 | + | 4 | D |
| 8 | 1 Y/F | WD | CI < 3mm. ON Inv –ve | 70 | + | 6 | E |
| 9 | 2Y/M | PD | CI>3mm. Inv In Anterior Portion of Sclera, ON Inv –ve. | 30 | + | 2 | E |
| 10 | 3Y/M | PD | CI <3mm.LamInv of ON +ve | 40 | ± | 2 | E |
| 11 | 11Mon/F | PD | CI <3mm. Lam Inv of ON –ve. | 40 | ± | 2 | E |
| 12 | 7Mon/F | WD | CI <3mm.Lam Inv of ON -ve | 70 | + | 6 | E |
| 13 | 2Y/M | MD | CI>3mm. Lam Inv & Post lam Inv of ON +ve | 90 | + | 6 | E |
| 14 | 71/2 Mon/M | WD | CI<3mm, Lam Inv & Post lam Inv of ON +ve. | 70 | + | 6 | E |
| 15 | 71/2 Mon/M | WD | CI<3mm, Lam Inv & Post lam Inv of ON +ve | 70 | + | 6 | E |

| **S.NO** | **AGE**  **/SEX** | **CLINICOPATHOLOGICAL Features** | | **CHGA** | | | |
| --- | --- | --- | --- | --- | --- | --- | --- |
|  |  | DIFFERENTIATION | INVASION STATUS | % POSITIVE  CELLS | STAINING  INTENSITY | SCORE |  |
| 1 | 3Y/F | PD | CI <3mm.ON Inv –ve. | 30 | ± | 1 | E |
| 2 | 2Y/F | MD | CI <3mm. ON Inv –ve. | 60 | + | 4 | E |
| 3 | 2Y/M | MD | CI<3mm. ON Inv –ve. | 70 | ± | 3 | E |
| 4 | 3Y/M | PD | CI <3mm.ON Inv –ve. . | 70 | + | 6 | E |
| 5 | 1 Y/F | WD | CI <3mm. ON Inv –ve. | 40 | ± | 2 | E |
| 6 | 2Y/F | PD | CI >3mm .ON Inv –ve. | 60 | + | 4 | E |
| 7 | 1Y/M | PD | CI >3mm. ON Inv –ve. | 20 | ± | 1 | D |
| 8 | 6Mon/F | WD | CI>3mm, tumor invading the Anterior, middle & posterior Scleral Inv +ve. lam & post lam Inv of ON +ve | 60 | + | 4 | E |
| 9 | 1Y/F | MD | Tumor Cells In AC, CI >3mm, Anterior& Middle Portion of Sclera Inv +ve. Lam & Post Lam Inv of ON +ve | 70 | ± | 3 | E |
| 10 | 2Y/M | MD | CI >3mm, tumor cells infiltrating Anterior Middle portion of Scleral Inv +ve , Lam Inv &Post lam Inv of ON +ve | 40 | ± | 2 | E |
| 11 | 11 Mon /F | PD | CI <3mm. Lam Inv of ON +ve | 50 | ± | 2 | E |
| 12 | 7Mon/F | WD | CI <3mm. Lam Inv of ON +ve | 60 | + | 4 | E |
| 13 | 1 Y/M | WD | CI <3mm, Lam & Postlam Inv of ON +ve | 60 | ± | 2 | E |
| 14 | 3Y/M | PD | CI <3mm, Lam & Post lam Inv of ON +ve | 80 | + | 6 | E |
| 15 | 7 Mon /M | WD | CI<3mm, Lam Inv&Post lam Inv of ON +ve | 30 | ± | 1 | E |

| **S.NO** | **AGE/SEX** | **CLINICOPATHOLOGICAL FEATURES** | | **MDK** | | | |
| --- | --- | --- | --- | --- | --- | --- | --- |
|  |  | DIFFERENTIATION | INVASION STATUS | %  POSITIVE CELLS | INTENSITY STAINING | SCORE | GROUP |
| 1 | 2Y/F | MD | CI <3mm.ON Inv –ve. | 30 | + | 2 | E |
| 2 | 6 Y/M | PD | CI >3mm.ON Inv –ve. | 20 | ± | 1 | E |
| 3 | 10 Mon/F | WD | CI>3mm.ON Inv –ve. | 60 | + | 4 | D |
| 4 | 1 Y/M | PD | CI >3mm.ON Inv –ve. | 60 | + | 4 | D |
| 5 | 7Mon/M | MD | CI <3mm .ON Inv –ve. | 60 | + | 4 | E |
| 6 | 1 Y/F | WD | CI < 3mm. ON Inv –ve. | 70 | + | 6 | E |
| 7 | 2Y/F | PD | CI >3mm .ON Inv –ve. | 60 | + | 4 | E |
| 8 | 3Y/M | PD | CI<3mm. Lam Inv of ON +ve | 70 | + | 6 | E |
| 9 | 71/2 Mon/M | WD | CI<3mm, Lam Invof ON +ve | 40 | ± | 2 | E |
| 10 | 3 Y/F | MD | CI < 3mm.Lam Inv of ON +ve | 60 | + | 4 | E |
| 11 | 7 Mon/F | WD | CI < 3mm. Lam Inv of ON +ve | 50 | + | 4 | E |
| 12 | 1Y/M | WD | CI <3mm,lam & post lam Inv of ON +ve | 70 | + | 6 | E |
| 13 | 2Y/M | MD | CI >3mm.lam & postlam Inv of ON +ve | 30 | + | 2 | E |
| 14 | 6 Mon/F | WD | CI>3mm, Lam & Post lam Inv of ON+ve | 90 | + | 6 | E |
| 15 | 11Mon/F | PD | CI<3mm, Lam & Post lam Inv of ON +ve | 30 | ± | 1 | E |

| **S.NO** | **AGE**  **/SEX** | **CLINICOPATHOLOGICAL FEATURES** | | **AHSG** | | | |
| --- | --- | --- | --- | --- | --- | --- | --- |
|  |  | DIFFERENTIATION | INVASION STATUS | % POSITIVE CELLS | INTENSITY | SCORE | GROUP |
| 1 | 2 YR/M | MD | CI <3mm, ON Inv –ve | 60 | ± | 2 | E |
| 2 | 10 Mon/F | WD | CI >3mm,ON Inv –ve | 40 | + | 4 | D |
| 3 | 1 Y/M | PD | CI <3mm ,ON Inv –ve | 70 | + | 6 | D |
| 4 | 2Y/F | PD | CI>3mm,ON Inv –ve | 60 | + | 4 | E |
| 5 | 1Y/F | WD | CI <3mm ,ON Inv –ve | 30 | + | 2 | E |
| 6 | 1 Y/F | WD | CI< 3 mm. ON Inv –ve. | 70 | + | 6 | E |
| 7 | 2 Yrs/M | PD | CI >3mm , Inv Into Anterior Border of Sclera, Middle Layer of Scleral Fibres | 30 | + | 2 | E |
| 8 | 2 Y/M | MD | Tumour Cells In The AC ,Iris, Stroma ,CI>3mm .Lam &Post lam Inv of ON +ve | 70 | + | 6 | E |
| 9 | 6 Mon/F | WD | CI>3mm, Lam & Post lam Inv of ON +ve. Tumor seen over The Iris Surface, Tumor Invading the Anterior, Middle & Posterior scleral Fibres. | 80 | + | 6 | E |
| 10 | 4 Y/F | PD | Tumor Cells In AC, CI <3mm, Anterior & Middle scleral Inv, ON Inv –ve. | 60 | + | 4 | E |
| 11 | 3 Y/M | PD | CI<3mm. lam Inv of ON +ve | 70 | + | 6 | E |
| 12 | 11 Mon/F | PD | CI<3mm, lam Inv of ON +ve | 50 | + | 4 | E |
| 13 | 7 Mon/F | WD | CI <3mm.Lam Inv of ON +ve | 60 | ± | 2 | E |
| 14 | 1YR/M | WD | CI <3mm, Lam & Minimal Post lam Inv of ON +ve | 20 | ± | 1 | E |
| 15 | 7 ½ Yrs/M | WD | CI <3mm,Lam & Post lam Inv of ON +ve | 60 | + | 4 | E |

| **S.NO** | **AGE/SEX** | **CLINIC-PATHOLOGICAL FEATURES** | | **RACGAP1** | | |
| --- | --- | --- | --- | --- | --- | --- |
|  |  | **DIFFERENTIATION** | **INVASION STATUS** | **%POSITIVE CELLS** | **STAINING**  **INTENSITY** | **SCORE** |
| 1 | 3 Y/M | MD | Foc RPE Inv, Pre-Lam & Lam ON Inv. | 90 | + | 9 |
| 2 | 3Y/M | PD | Foc RPE Inv, Pre-Lam & Lam ON Inv. | 50 | + | 6 |
| 3 | 7Mon/ M | MD | Foc CI<3mm, Pre-Lam ON Inv . | 70 | + | 9 |
| 4 | 7 Y/M | WD | Foc CI<3mm, Pre-Lam ON Inv. | 60 | + | 6 |
| 5 | 2 Y/F | PD | Foc RPE Inv. CI >3mm , Pre-Lam ON Inv. | 70 | + | 9 |
| 6 | 2Y/F | PD | Pre-Lam ON Inv. | 70 | + | 9 |
| 7 | 10 Mon/F | WD | CI >3mm. | 60 | + | 6 |
| 8 | 2 Y/M | MD | CI >3mm. Prelam, Lam & Postlam ON Inv. | 80 | + | 9 |
| 9 | 3 Y/M | PD | Foc Retinoma , No CI & ON Inv. | 30 | +/- | 2 |
| 10 | 4Y/M | MD | Mild Pre-Laminar ON Inv. | 40 | + | 3 |
| 11 | 2Y/M | PD | No CI & ON Inv | 50 | + | 6 |
| 12 | 1Y/M | WD | No CI & ON Inv | 70 | + | 9 |
| 13 | 1 Y/M | WD | No CI & ON Inv | 70 | + | 9 |
| 14 | 7Mon/M | WD | No CI & ON Inv | 60 | +/- | 4 |
| 15 | 10Mon/F | MD | No CI & ON Inv | 40 | + | 3 |

Table S2 Clinicopathological features of RB primary tumors used in the study

| **S.NO** | **AGE/SEX** | **Group/laterality** | **Clinicopathological parameters** |
| --- | --- | --- | --- |
| 1 | 1 Year/Male | Group E, OS | MD, Tumor cells are invading the anterior chamber, iris stroma and ciliary body. There is choroidal invasion >3mm. Tumor cells are invading the anterior and middle portion of scleral fibers, There is pre-laminar , laminar &post laminar invasion of optic nerve by the tumor cells. Surgical end of optic nerve is free from tumor cells. |
| 2 | 1 Year /Male | Group E, OD | MD, Tumor cells are invading the anterior chamber, There is choroidal invasion >3mm. Tumor cells are invading the anterior and middle portion of scleral fibers. There is no Optic nerve invasion by tumor cells |
| 3 | 3 Years /Female | Group E ,OD | MD, With Focal retinoma with Choroidal Invasion 3mm. There is no Optic nerve invasion by tumor cells |
| 4 | 4 Years/Male | Group E ,OS | WD, Choroidal Invasion >3mm. There is only pre-laminar Invasion of optic nerve by tumor cells. |
| 5 | 6 Months /Female | Group E ,OD | WD, Tumor seen over the iris surface Choroidal Invasion >3mm, Pre-laminar, Laminar & Post-laminar Invasion of Optic Nerve is present. Tumor invading the Anterior, middle and posterior scleral fibers. Surgical end of optic nerve is free from tumor cells. |
